# The influence of perceived social support on anxiety among international students: the mediating role of communicative adaptability

**DOI:** 10.3389/fpsyg.2025.1498261

**Published:** 2026-01-12

**Authors:** Lili Song, Chundi Peng, Yang Hai, Jingya Wang, Jiyang Zhao

**Affiliations:** 1 College of International Education of Harbin Medical University, Harbin, Heilongjiang, China; 2 School of Mental Health, Wenzhou Medical University, Wenzhou, China

**Keywords:** anxiety, perceived social support, communicative adaptability, international students, mental health

## Abstract

**Background:**

Anxiety is one of the psychological problems that cannot be ignored among many international students. Long-term anxiety has a significant negative impact on the social life and academic achievement of international students. Without timely intervention, it may gradually escalate, induce extreme high-risk behaviors, and seriously threaten the life, health and safety of international students. This study aims to investigate the current situation of anxiety among international students, analyze the influence of perceived social support on anxiety among international students, and explore the mediating role of communicative adaptability.

**Methods:**

This study was conducted in June 2024, and a convenience sampling method was used to investigate 198 international students in a university in Harbin. Measurements included a general demographic questionnaire, Depression Anxiety Stress Scale, Multidimensional Scale of Perceived Social Support, and Communicative Adaptability Scale. In this study, SPSS27.0 software was used to conduct descriptive statistical analysis, t test and variance analysis, correlation analysis and regression analysis. Meanwhile, PROCESS plug-in in SPSS27.0 software was used to test the mediation model.

**Results:**

The results of correlation analysis showed that perceived social support was positively correlated with communicative adaptability (*r* = 0.389, *p* < 0.01) and negatively correlated with anxiety (*r* = −0.325, *p* < 0.01). Communicative adaptability was negatively correlated with anxiety (*r* = −0.250, *p* < 0.01). The mediating effect of communicative adaptability was significant in perceived social support on anxiety (95% CI: −0.131 to −0.013).

**Conclusion:**

Providing more adaptive and targeted social support and cultural adaptation activities may effectively alleviate the anxiety level of international students, and thus maintain and improve the mental health of international students.

## 1 Introduction

Since the 21st century, globalization has accelerated the development of international educational exchanges, with the number of international students steadily increasing and their backgrounds becoming increasingly diverse. However, students often encounter language barriers, culture shock, and difficulties in social integration during cross-cultural studies, and these stressors significantly impact their mental health (Deb and Miller, 2017; Sirin et al., 2013). Particularly for first-time overseas students, substantial cultural differences, language barriers, and the intertwined emotions of homesickness and loneliness make them more susceptible to psychological issues such as anxiety and depression (Wang et al., 2015). In severe cases, self-harm or suicidal behavior may occur (Chou et al., 2011), not only undermining academic performance and personal development but also posing potential threats to campus stability and social harmony (Araujo et al., 1999).

Anxiety is one of the psychological issues that cannot be ignored among many international students. As an instinctive emotional response mechanism, anxiety serves an adaptive functions within a moderate range. However, excessive anxiety may lead to emotional or physical illness. If anxiety symptoms persistently impair daily functioning and lead to abnormal behavior, it constitutes an anxiety disorder requiring intervention and treatment (Dean, 2016). In the face of the challenge of anxiety, international students showed a significant feature, that is, a low willingness to actively seek psychological treatment. Skromanis et al. (2018) found that compared to Australian domestic students, male international students are more vulnerable to mental health issues and significantly less active in seeking psychological assistance. However, long-term anxiety has a significant negative impact on international student’s social lives and academic achievements. Without timely intervention, anxiety may escalate progressively, triggering extreme high-risk behaviors that severely threaten students’ health and personal safety (Pillay, 2021). Therefore, it is particularly important to explore the mechanism of how to effectively intervene and alleviate the anxiety of international students.

The main effects model of social support emphasizes its impact on individual’s physical and mental health (Cohen and Wills, 1985). This model indicates that social support enhances individual adaptability, effectively alleviates stress, and thereby improves physical and mental health. In addition, studies have further confirmed that perceived social support directly predicts negative emotions and alleviates negative emotions to a certain extent (Li et al., 2016). Perceived social support refers to the degree to which an individual feels understood, supported and respected in social interactions, along with the resulting emotional satisfaction and experiences (Dahlem et al., 1991). Studies have shown that intrinsic perceived social support is an important factor affecting college students’ anxiety (Lee et al., 2013). As a positive variable, perceived social support can effectively reduce anxiety (Gündüz et al., 2018). Its positive effect is that when international students face challenges or stress, they can rely on this sense of subjective support to mobilize internal resources and enhance coping abilities, thereby alleviating anxiety triggered by various stressors.

Cross-cultural adaptation refers to profound changes triggered when individuals or groups from different cultures interact, manifesting across emotional, behavioral, and cognitive dimensions (Searle and Ward, 1990). For this unique and highly sensitive group of international students, adaptive capacity is a critical factor in ensuring academic success, social interaction, and psychological well-being. Kim’s (2003) Integrative Communication Theory reveals that cross-cultural adaptation is a dynamic dialectical process of “stress-adaptation-growth,” emphasizing individuals’ functional adaptability and psychological well-being in new environments. Within this adaptation process, challenges such as language barriers and cultural custom differences frequently pose difficulties for international students, often leading to adaptation dilemmas (Chung and Epstein, 2014). Such adaptive issues are regarded as important mediating variables detrimental to international students’ mental health (Berry, 2005). Academic consensus holds that high-intensity cultural adaptation stress often exerts significant negative effects on individual psychological states, with anxiety being a common emotional response. Against this backdrop, the perceived social support experienced by international students during their overseas lives plays a crucial role. Social support not only provides emotional comfort and psychological security but also helps alleviate feelings of uncertainty and loneliness stemming from cross-cultural communication barriers (Albrecht and Adelman, 1984; Adelman, 1988), thereby effectively reducing anxiety experiences. This study proposes the following hypothesis: Communicative adaptability mediates the effect of perceived social support on anxiety among international students.

Therefore, this study aims to investigate the current status of anxiety among international students, analyze and understand the perceived social support on anxiety, and explore the mediating role of communicative adaptability. The study objectives is to provide more precise and effective strategic suggestions for improving the anxiety of international students, thereby enhancing the overall mental health, building a solid psychological defense for their overseas studies, and promoting personal growth and continuous progress.

## 2 Methods

### 2.1 Participants and procedures

This study was conducted in June 2024. A convenience sampling method was adopted to investigate all the international students on campus in a certain university in Harbin. The inclusion criteria for the research subjects are: regular international students currently enrolled, at least 18 years old and willing to participate. Exclude exchange students and those who are unable to understand the questionnaire due to language barriers. A total of 230 questionnaires were sent out. After careful screening and sorting (excluding those with incomplete responses, regular responses, and those that failed the lie detector test), 198 valid questionnaires were successfully recovered, with an effective rate of 86.08%. The study was approved by the Ethics Committee of Harbin Medical University. In the recruitment and data collection stage of participants, we strictly implemented the principle of informed consent.

### 2.2 Measures

Demographic information was collected, including gender, parental marital status, parental education level and physical activity status.

Depression Anxiety Stress Scale (DASS) consists of 3 subscales, with a total of 21 items, respectively to investigate the degree of individuals’ experience of depression, anxiety, stress and other negative emotions (Lovibond and Lovibond, 1995). A 4-point scale of 0 to 3 points is used to score, 0 means “Did not apply to me at all,” 3 means “Applied to me very much, or most of the time.” The sum of the seven scores for each subscale is multiplied by 2 to give the subscale score, with higher scores indicating higher levels of depression, anxiety, or stress. Since this study primarily focuses on the anxiety levels of international students, the anxiety subscale was selected. In this study the total questionnaire Cronbach’s alpha coefficient was 0.955, and the Cronbach’s alpha coefficient for anxiety was 0.860. In previous studies, the scale has been demonstrated to be suitable for diverse sample populations, including university students from different countries and international student groups, exhibiting good cross-cultural applicability (Moussa-Chamari et al., 2024; Slaughter et al., 2023).

Multidimensional Scale of Perceived Social Support (MSPSS) contains 12 items (Zimet et al., 1988), which are divided into three subscales: significant others, family and friend support, and is scored on a 7-point scale (1 means “very strongly disagree,” 7 means “very strongly agree”). The Cronbach’s alpha coefficient was 0.949. The MSPSS scale has been applied to college students with international student status in previous studies, confirming its applicability within this population (Bokszczanin et al., 2023).

Communicative Adaptability Scale (CAS) consists of 30 items (Worthington and Bodie, 2017), including six dimensions: Social Composure, Social Confirmation, Social Experience, Appropriate Disclosure, Articulation and Wit. The rating scale uses a 5-point scale, with 1 indicating “never true of me” and 5 indicating “always true of me,” and some items require a reverse scoring. The Cronbach’s alpha coefficient was 0.872. The applicability of the CAS scale has been validated by previous studies and is suitable for the international student population involved in this research (Duran, 1983).

### 2.3 Statistical methods

This study employed SPSS 27.0 software for statistical analysis, utilizing two-tailed tests with *p* < 0.05 indicating statistically significant differences. Specific methods included descriptive statistics, *t*-tests, analysis of variance (ANOVA), correlation analysis, and regression analysis. Prior to mediation analysis, all continuous variables underwent centering to eliminate multicollinearity. The mediation model was tested using Model 4 within the PROCESS plugin.

## 3 Results

### 3.1 Demographics

The study primarily involved Asian international students, with India (70 participants), Bangladesh (25 participants), and Indonesia (16 participants) as the main countries of origin. The sample also encompassed a diverse group from 28 countries across the Americas, Europe, and other regions. The majority of participants are enrolled in six-year degree programs, and their native languages are the official or primary languages of their respective countries. There were 105 male students and 93 female students in this study. 6.06% of the students’ parents are divorced; 55.05% of the students’ fathers have a bachelor’s degree or above; 40.91% of the students’ mothers have a bachelor’s degree or above; 38.89% of the students often do sports. See Table 1 for details.

**Table 1:** The basic information of international students in our research (*n* = 198).

Variable	Number of people	Percentage (%)
Gender		
Male	105	53.03%
Female	93	46.97%
Parental divorce or not		
Yes	12	6.06%
No	186	93.94%
Father’s years of schooling		
less than 5 years	9	4.55%
5–9 years (junior high school degree)	10	5.05%
10–12 years (high school education)	28	14.14%
13–16 years (undergraduate / other)	42	21.21%
16 years (bachelor degree or above)	109	55.05%
Mother’s years of schooling		
less than 5 years	18	9.09%
5–9 years (junior high school degree)	15	7.58%
10–12 years (high school education)	44	22.22%
13–16 years (undergraduate / other)	40	20.20%
16 years (bachelor degree or above)	81	40.91%
Do you often have sports		
Once in a while	62	31.31%
Less	59	29.80%
Often	77	38.89%

### 3.2 Scores of anxiety, perceived social support and communicative adaptability of international students

In this study, 38.4% of international students experience anxiety. The students’ anxiety scores were (7.67 ± 8.79), perceived social support scores were (5.17 ± 1.53), and communicative adaptability scores were (100.09 ± 19.24).

### 3.3 Correlation analysis

The results showed that perceived social support was positively correlated with communicative adaptability (*r* = 0.389, *p* < 0.01), and negatively correlated with anxiety (*r* = −0.325, *p* < 0.01). There was a significant negative correlation between communicative adaptability and anxiety (*r* = −0.250, *p* < 0.01). See Table 2 for details.

**Table 2:** The correlation of perceived social support, communicative adaptability and anxiety.

Scales	*M* ± SD	1	2	3
Perceived social support	5.17 ± 1.53	1		
Communicative adaptability	100.09 ± 19.24	0.389^**^	1	
Anxiety	7.67 ± 8.79	−0.325^**^	−0.250^**^	1

**p < 0.01.

### 3.4 The relationship between perceived social support and anxiety: the mediating role of communicative adaptability

**Figure 1:**
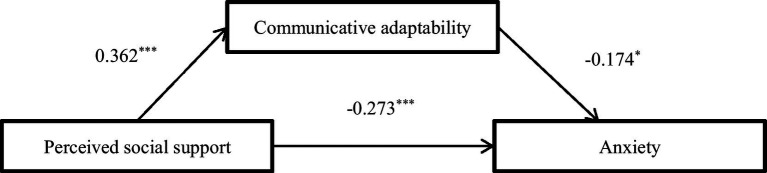
The mediation model of perceived social support → anxiety. ^*^p < 0.05, ^**^p < 0.01, ^***^p < 0.001.

The results showed that perceived social support was negatively correlated with anxiety (*β* = −0.273, *p* < 0.05), communicative adaptability was negatively correlated with anxiety (*β* = −0.174, *p* < 0.05), and perceived social support was positively correlated with communicative adaptability (*β* = 0.362, *p* < 0.05). After controlling for demographic variables including gender, parental marital status, parental education level, and physical activity status, mediation analysis revealed that the mediation effect was significant (95% CI: –0.131 to –0.013). The direct effect of perceived social support on anxiety was also significant (95%CI: −0.418 to −0.128). It is suggested that the influence of perceived social support on anxiety is mediated by direct and indirect communicative adaptability pathways. Relevant data are detailed in Tables 3, 4, with the mediation model illustrated in Figure 1.

**Table 3:** Mediation analysis of communicative adaptability on the relationship between perceived social support and anxiety.

Variable	*B*	SE	*T*	*p*
Perceived social support on anxiety	−0.336	0.069	−4.860	<0.001
Perceived social support on communicative adaptability	0.362	0.066	5.501	<0.001
Communicative adaptability on anxiety	−0.174	0.075	−2.320	0.021
Perceived social support on anxiety	−0.273	0.074	−3.709	<0.001

**Table 4:** Bootstrap results for the mediation analysis.

Effect	Effect size	SE	LL95%CL	UL95%CL
Total effect	−0.336	0.069	−0.472	−0.200
Direct effect	−0.273	0.074	−0.418	−0.128
Indirect effect	−0.063	0.031	−0.131	−0.013

## 4 Discussion

In this study, 38.4% of international students have anxiety, previous studies have also pointed out that there is widespread anxiety among international students (Mahihu, 2020; Wang et al., 2023). The reason can be attributed to the fact that some students are immersed in a new cultural environment, where they encounter different cultural and social backgrounds, which often hinders their rapid integration into the local society. This shift requires a redefinition of their identity and values, leading to deep internal reconstruction, often accompanied by conflict, which greatly exacerbates their state of anxiety. In addition, academic pressure becomes a key factor to be concerned about; The high academic demands of studying abroad, with homework, exams and attendance standards often extremely rigorous, further exacerbate anxiety levels.

This study examines the relationship between international students’ perceived social support and anxiety, revealing a significant negative correlation between the two. Specifically, perceived social support serves as a negative predictor of anxiety within this population. These findings hold significant implications for understanding the mental health challenges faced by international students. Upon first arriving in a foreign country, international students often experience fear and unease when confronting unfamiliar academic and living environments. Perceived social support plays a crucial role in facilitating their adaptation process. It encompasses the understanding, respect, and assistance individuals receive from others—forming a positive emotional experience that enhances psychological resilience while significantly alleviating ruminative thinking (Abela et al., 2004; Flynn et al., 2010). Additionally, perceived social support can motivate international students to engage in social interactions and build meaningful relationships, thereby reducing anxiety levels. Over time, perceived social support exerts a profound impact on mental health: it not only fosters positive self-perception, enhances self-confidence and self-esteem, but also facilitates emotional expression and regulation (Tabatabaei et al., 2018; Wirtz et al., 2006). This psychological reinforcement mechanism helps international students better adapt to life abroad and reduces psychological issues stemming from maladjustment.

Communicative adaptability can negatively predict the anxiety of international students. Social anxiety is a prevalent psychological issue among international students, which is mainly manifested as nervousness, unease and fear of rejection in social situations. Group anxiety theory holds that when individuals interact with members outside their own group, they are more prone to anxiety reactions due to concerns about potentially triggering negative evaluations or behavioral consequences (Fang et al., 2016). Insufficient language proficiency and cultural differences may exacerbate this self-doubt and psychological burden. Furthermore, strong communication adaptability not only facilitates social integration but also positively impacts academic performance. By interacting with local students, international students can access learning resources, master efficient study methods, and adjust their learning strategies based on feedback, thereby alleviating anxiety stemming from academic pressure (Finch and Vega, 2003; Mak and Kim, 2011; Durlak and Weissberg, 2011). Furthermore, gender exerts a significant influence on an individual’s cross-cultural adaptation. Berry’s research indicates that male immigrants typically demonstrate stronger psychological adaptation, while females exhibit relative advantages in sociocultural adaptation, reflecting the differential impact of gender factors across different dimensions of adaptation (Berry, 2005).

This study found that communicative adaptability mediates the relationship between perceived social support and anxiety. Specifically, high anxiety among international students is often closely linked to their perceived lack of social support, which further intensifies the impact of communicative adaptability on anxiety. Perceived social support provides individuals with both informational and emotional assistance. Particularly among friends from the same cultural background who share similar experiences, their targeted assistance fosters a sense of belonging, effectively alleviating anxiety (Adelman, 1988). Simultaneously, perceived social support helps reduce psychological stress, boost self-confidence, and promote cultural understanding, thereby enhancing communicative adaptability (Kim and Stoner, 2008). According to cross-cultural adjustment theory, international students’ cultural adaptation is a dynamic process involving the modification of existing cultural patterns and the internalization of new cultural norms (Berry, 1980). During this process, students can effectively reduce barriers in cross-cultural interactions and enhance social identification and belonging by consciously adjusting verbal or nonverbal behaviors and adopting strategies such as convergence and accommodation. Such behaviors not only facilitate deeper social support and the formation of high-quality cross-cultural friendships but also create more opportunities to employ adaptive emotional regulation strategies (e.g., emotional seeking, cognitive coping) through expanded social support networks. Ultimately, this positive adaptation process significantly alleviates international students’ anxiety by enhancing their sense of self-efficacy (Schimmack, 2005). Therefore, it can be argued that perceived social support indirectly reduces anxiety among international students by promoting communicative adaptability. This mediating effect reveals the profound impact of perceived social support on international students’ mental health and provides an effective pathway for alleviating their anxiety.

Based on the research findings, the following interventions and policy recommendations are proposed to further enhance international students’ cross-cultural adaptation: First, it is essential to integrate faculty, peer, and volunteer resources to establish a sustained and effective support network, ensuring international students receive timely, multi-source understanding and assistance in both academic and daily life. Second, actively create opportunities for cross-cultural interaction. Organize and encourage international students to participate in regular cultural exchange activities such as local festivals, cultural lectures, and language corners. This promotes deeper engagement with local residents, thereby enhancing their understanding of and identification with the host country’s culture. Additionally, universities and relevant institutions must prioritize the critical adaptation phase during the initial enrollment period. Specialized cultural adaptation training courses should be offered to international students, systematically introducing local social norms, behavioral customs, and interpersonal etiquette. This approach will mitigate feelings of cultural shock and accelerate their integration process.

### 4.1 Limitations and suggestions for future research

This study is mainly based on self-reported questionnaires, which leads to a certain subjectivity of the results, limiting the objectivity and accuracy of the conclusions. Second, because this study was cross-sectional, it is not possible to directly determine cause and effect. To gain a deeper understanding of the mechanisms affecting anxiety conditions, future research could use longitudinal data to explore causality. Furthermore, we did not control for potential confounding variables, such as age, academic level, socioeconomic background, or local social networks, which might influence the observed outcomes. Lastly, the generalizability of our results is constrained by the single-center sampling strategy, and future studies involving multi-center or diverse populations are warranted.

## 5 Conclusion

In conclusion, this study reveals the close correlation between international students’ anxiety and perceived social support, especially emphasizing the mediating role of communicative adaptability. The high anxiety level of international students may be due to the fact that international students feel less social support, which intensifies the influence of adaptability on anxiety. Therefore, providing more adaptive and targeted social support and cultural adaptation activities may effectively alleviate the anxiety level of international students, and thus maintain and improve the mental health of international students.

## Data Availability

The datasets presented in this article are not readily available because the data that support the findings of this study are available from the corresponding author upon reasonable request. Requests to access the datasets should be directed to zhaojy@hrbmu.edu.cn.
